# The outcome of lateral rectus extirpation in a case of traumatic third cranial nerve palsy: A case report

**DOI:** 10.1016/j.ijscr.2025.111887

**Published:** 2025-09-02

**Authors:** Shaimaa M. Alrefaie, Rahaf A. Afandi, Fawzia M. Alhaimi

**Affiliations:** a Pediatric Ophthalmology and Strabismus Division, King Khalid Eye Specialist Hospital, Riyadh, Saudi Arabia; b Ophthalmology Residency Program, Ohud Hospital, Madinah, Saudi Arabia

**Keywords:** Third cranial nerve palsy, Oculomotor nerve palsy, Diplopia, Ptosis, Surgical management, Lateral rectus extirpation

## Abstract

**Introduction and clinical importance:**

To present a case of traumatic third cranial nerve palsy and discuss the management challenges associated with this condition.

**Case presentation:**

A 27-year-old male patient was referred to our hospital following a road traffic accident that resulted in multiple injuries, including traumatic brain injury, orbital injury. The patient presented with left complete upper lid ptosis, a fixed dilated pupil, and restricted extraocular muscle movements in the left eye except abduction with large exotropia >90 PD and hypotropia 25 PD diagnosed as left oculomotor nerve palsy. Imaging studies confirmed the presence of multiple orbital fractures.

**Clinical discussion:**

Oculomotor nerve palsy is a common and significant clinical finding, accounting for 30 % of all cranial nerve palsies. Third nerve palsy presents the greatest challenge, as it affects four of the six extraocular muscles, necessitating a different treatment approach. Managing patients with this condition is challenging, as the outcomes of various surgical techniques have generally been viewed as unsatisfactory. The primary goal of surgery is to achieve good ocular alignment in the primary position, but it is rarely effective in restoring measurable binocular function.

**Conclusion:**

This case report highlights the complex nature of managing traumatic third cranial nerve palsy, which requires a comprehensive approach to address the patient's visual and functional impairments. Further research and advancements in surgical techniques are needed to improve outcomes for patients with this condition.

## Highlights

• Oculomotor nerve palsy is a common and significant clinical finding, accounting for 30 % of all cranial nerve palsies.
• Managing patients with this condition is challenging, as the outcomes of various surgical techniques have generally been viewed as unsatisfactory.
• The complex nature of treatment traumatic third cranial nerve palsy, requires a comprehensive approach to address the patient's visual and functional impairments.

## 1 Introduction

Oculomotor cranial nerve palsy is a common and significant clinical finding, accounting for 30 % of all cranial nerve palsies [1,2]. Of the three ocular motor nerve palsies, the third nerve palsy presents the greatest challenge to clinical management because it affects four of the six extraocular muscles, necessitating a different treatment strategy [3]. Its major features include diplopia and ptosis resulting from the involvement of the superior rectus, inferior rectus, medial rectus, and levator palpebrae superioris muscles. Additionally, through its parasympathetic fibers, it innervates the ciliary muscle, the annular portion, and the iris sphincter, which, if compromised, results in a fixed dilated pupil [1].

Third nerve palsies can be classified as either congenital or acquired, total or partial, isolated, or associated with other nerve palsies [3]. In complete palsy, the eye is in exotropia, hypotropia, and cyclotorsion with restricted adduction, elevation, and depression. Pupillary mydriasis is particularly related to cases of acquired palsies secondary to trauma or space-occupying lesions [4]. In children, the most common etiologies are congenital (43 %), traumatic (20 %), inflammatory (13 %), and aneurysmal (7 %). In contrast, the most common causes in the adult population are vasculopathic disorders (diabetes mellitus, hypertension), aneurysms, and trauma [3].

Managing patients with oculomotor nerve palsy is one of the most challenging issues addressed by ophthalmologists, as the outcomes of several surgical techniques reported in the past have generally been viewed as unsatisfactory [5]. Multiple studies have agreed that the major goal of surgery is achieving good ocular alignment in the primary position (PP) [3,[6], [7], [8]]. While sometimes successful in cosmetically realigning the eyes, surgery is rarely effective in restoring measurable binocular function [6]. Therefore, we report the outcome of lateral rectus muscle extirpation in a case of traumatic third cranial nerve palsy. This work has been reported in line with the SCARE criteria [9].

## 2 Case presentation

A 27-year-old male patient was referred to our hospital in January 2017. The patient had been involved in a road traffic accident and was admitted to the intensive care unit (ICU) at the referring hospital.

**Fig. 1:**
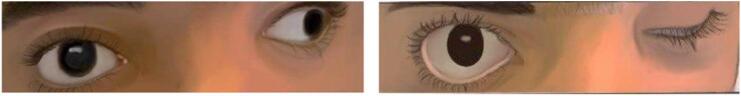
Left eye complete ptosis and exotropia and hypotropia in the primary position.

Examination of patient revealed complete left upper lid ptosis. Visual acuity was 20/25 in the right eye and no light perception (NLP) in the left eye. The left pupil was fixed and dilated with +3 relative afferent pupillary defect (RAPD). Extraocular motility of the left eye was restricted in all gazes except for abduction. The patient had left eye exodeviation (XT) more than 90 prism diopters (PD) in the primary position and a left hypotropia 16 PD as demonstrated in the schematic digital illustration (Fig. 1).

Slit lamp examination showed a central ring corneal scar in the left eye with mild corneal superficial punctate keratitis (SPKs) and a poor view of the fundus after dilation. A B-scan ultrasound revealed a normal posterior pole.

A CT scan of the brain and orbit was demonstrating multiple left orbital fractures more in medial wall and sparing superior wall. This was associated with left lateral rectus entrapment and lateral globe deviation.

The patient was diagnosed with a complete left eye traumatic third cranial nerve palsy, left traumatic optic neuropathy, and enophthalmos. Other cranial nerves were intact.

The patient was scheduled for left eye medial rectus resection by 8 mm plus lateral rectus extirpation.

During strabismus surgery. The lateral rectus was found in normal anatomy and not entrapment and it was hooked by two muscle hooks as far as possible then after securing the muscle we hold it with opticlamp afterward we cut a large piece of the muscle as large as possible, then we did maximum cautery in the original site then we let the muscle to go back into its sac and then we suture the sac.

After two weeks post-operatively, the patient came for follow-up and was seen in good condition with a residual XT of 16 PD and mild duction movements.

The ptosis repair surgery (frontalis sling) for left ptosis was done in November 2020.

In 2021, the patient was interested in another strabismus surgery to improve the remaining XT 30 PD and hypotropia 16 PD. So left MR re-resection 5 mm and left superior oblique tenectomy was done.

**Fig. 2:**
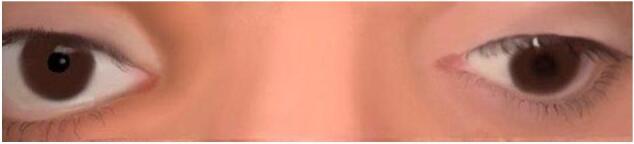
Post operatively, the patient had exodeviation of 20 PD, Left eye.

After the surgery, the patient still had residual exotropia of 20 PD in the left eye but it was cosmetically acceptable (Fig. 2).

## 3 Discussion

Third cranial nerve palsy presents a significant challenge in ophthalmic practice, accounting for approximately 30 % of all ocular motor nerve palsies [10,11]. The management of this condition is particularly complex due to its impact on multiple extraocular muscles and the upper eyelid, often resulting in a combination of strabismus, ptosis, and pupillary abnormalities [12].

In our case report, we present a patient with traumatic complete third nerve palsy, which aligns with findings from larger studies. Bagheri et al., in their 10-year retrospective study, found that trauma was the most common etiology of third nerve palsy, accounting for 50 % of cases [13]. This high percentage of traumatic cases may be attributed to the fact that such studies are often conducted in tertiary referral centers, where more complex cases are likely to be seen.

The surgical management of third nerve palsy remains a subject of debate, with various techniques proposed over the years. The primary goal of surgery is to achieve good ocular alignment in the primary position, although restoration of binocular function is rarely achieved [14]. In our case, we opted for lateral rectus extirpation combined with maximum medial rectus resection, which provided satisfactory results in terms of reducing exotropia.

This approach differs from some of the more recently proposed techniques. For instance, Saxena et al. reported positive outcomes with medial transposition of a split lateral rectus muscle augmented with fixation sutures [15]. Their technique achieved a mean correction of 51.5 prism diopters (PD) of exotropia and 10.5 PD of hypotropia. Similarly, Gokyigit et al. described a technique of medial transposition of a split lateral rectus muscle, which resulted in a mean correction of 63.6 PD of exotropia [16].

Another approach that has been explored is the transposition of the superior oblique muscle. Metz and Yee reported on this technique, which aims to convert the superior oblique into an adductor, potentially improving both horizontal and vertical alignment [17]. However, this technique is not widely adopted due to its technical complexity and unpredictable results.

Our choice of lateral rectus extirpation, while less commonly reported in recent literature, proved effective in our patient. This highlights the importance of tailoring the surgical approach to each individual case, considering factors such as the degree of muscle paralysis, the extent of deviation, and the presence of associated injuries.

The management of ptosis in third nerve palsy cases presents an additional challenge. In our patient, frontalis sling surgery was performed to address the ptosis. This approach is often favored in cases of poor levator function, as is common in third nerve palsy [12]. However, the risk of exposure keratopathy must be carefully considered, especially in cases with poor Bell's phenomenon, as was the case with our patient.

It's worth noting that despite advances in surgical techniques, the management of complete third nerve palsy remains challenging, with many patients requiring multiple surgeries to achieve satisfactory results. Our case demonstrates the potential for good outcomes with a combination of strabismus and ptosis surgeries but also highlights the need for long-term follow-up and potential revisions.

In conclusion, this case report underscores the complex nature of managing traumatic third cranial nerve palsy and the importance of a comprehensive, individualized approach. While various surgical techniques have been proposed, the choice of procedure should be based on careful consideration of each patient's specific presentation and needs. Further research into novel surgical techniques and long-term outcomes is needed to continue improving our management of this challenging condition.

## Consent

Informed consent for the use of clinical images was discussed with the patient; however, the patient declined to provide authorization. Accordingly, all clinical photographs were omitted, and schematic digital illustrations were generated and utilized in place of original patient images to ensure confidentiality and compliance with ethical standards.

## Ethical approval

The study approved by the institutional review board (IRB) committee at King Khaled Eye Specialist Hospital (KKESH).

Study reference/number: RD/26001/IRB/0367-24

## Funding

King Khaled Eye Specialist Hospital (KKESH).

## Author contribution

Shaimaa M. Alrefaie: Study concept and design, writing the manuscript, data collection.

Rahaf A. Afandi: Writing the manuscript, data analysis, journal instructions.

Fawzia M. Alhaimi: Study concept and design, data interpretation, modification of the manuscript.

## Guarantor

Shaimaa M. Alrefaie.

Rahaf A. Afandi.

Fawzia M. Alhaimi.

## Research registration number

None.

## Conflict of interest statement

No conflicting relationships exist for any author. However, the authors would like to extend their gratitude to King Khaled Eye Specialist Hospital (KKESH) and the Ethics Committee for their valuable support, funding and approval for conduct of this research.
